# Supplementary material for: The Effect of Sows’ and Piglets’ Behaviour on Piglet Crushing Patterns in Two Different Farrowing Pen Systems
Source: Animals (Basel). 2019 Aug 7;9(8):538. doi: 10.3390/ani9080538 (PMC6719171; doi:10.3390/ani9080538)
Supplement: Supplementary file 1 [file animals-09-00538-s001.pdf]

Table S1. Ethogram of the recorded sow behavior during 72 hours postpartum and during piglet crushing.

body posture	standing	sow solely touches the ground with three or four limbs (except snout)
	sitting	weight-bearing of the forelimbs, but not of the hind limbs. Ham touches ground.
	sternal recumbency	sow touches the ground with the belly side/underside of the body, but not with her body sides. No weight-bearing of any limb.
	lateral recumbency	sow touches the ground with the whole of one body side, not weight-bearing of any limb and at least three limbs are next to the body of the sow.
body posture changes (piglet crushing)	lay down in sternal recumbency	physiological and controlled lying down from a standing or sitting position in sternal recumbency
	lay down in lateral recumbency	physiological and controlled lying down from a standing or sitting position in sternal recumbency, followed by an immediate change from sternal to lateral recumbency
	"flopping" in lateral recumbency	uncontrolled fall down of the sow from a standing or sitting position in lateral recumbency
	lateral recumbency to sternal recumbency	transition from lateral recumbency to sternal recumbency
	sternal recumbency to lateral recumbency (central position)	transition from a central sternal recumbency to lateral recumbency
	sternal recumbency to lateral recumbency (same side)	transition from an asymmetric sternal recumbency (a distinction can be made between udder side and back side) to lateral recumbency in direction of the back side
	sternal recumbency to lateral recumbency (swap side)	transition from an asymmetric sternal recumbency (a distinction can be made between udder side and back side) to lateral recumbency in direction of the udder side
	sternal recumbency to sternal recumbency	position correction without changing the body posture sternal recumbency

Table S2. Ethogram of the recorded piglet behavior in the first 72 hours postpartum and during piglet crushing.

location	creep area	$\geq$ 50% of the litter in the creep area
	pen	> 50% of the litter in the pen
active / inactive	active	> 50% of the piglets in the pen are active (standing, sitting, sucking)
	inactive	$\geq$ 50% of piglets in the pen are inactive (lying, except sucking)
resting behavior next to the sow during rolling	no resting behavior	no piglets rest next to sow\`s body immediate to the rolling event
	resting behavior	one or more piglets rest next to sow\`s body immediate to the rolling event
